# Genetic structure of *Plasmodium falciparum *field isolates in eastern and north-eastern India

**DOI:** 10.1186/1475-2875-6-60

**Published:** 2007-05-21

**Authors:** Hema Joshi, Neena Valecha, Anju Verma, Asha Kaul, Prashant K Mallick, Sneh Shalini, Surendra K Prajapati, Surya K Sharma, Vas Dev, Sukla Biswas, Nutan Nanda, MS Malhotra, Sarala K Subbarao, Aditya P Dash

**Affiliations:** 1 National Institute of Malaria Research (ICMR), 22-Sham Nath Marg, Delhi-110 054, India; 2 National Institute of Malaria Research, Field Unit IDVC, Sector 5, Rourkela-769 002, Orissa, India; 3 National Institute of Malaria Research, Field Unit IDVC, PO Sonapur, District Kamrup, Assam-782 402, India; 4 Indian Council of Medical Research, V. Ramalingaswami Bhawan, Ansari Nagar, New Delhi 110029, India

## Abstract

**Background:**

Molecular techniques have facilitated the studies on genetic diversity of *Plasmodium *species particularly from field isolates collected directly from patients. The *msp-1 *and *msp-2 *are highly polymorphic markers and the large allelic polymorphism has been reported in the block 2 of the *msp-1 *gene and the central repetitive domain (block3) of the *msp-2 *gene. Families differing in nucleotide sequences and in number of repetitive sequences (length variation) were used for genotyping purposes. As limited reports are available on the genetic diversity existing among *Plasmodium falciparum *population of India, this report evaluates the extent of genetic diversity in the field isolates of *P. falciparum *in eastern and north-eastern regions of India.

**Methods:**

A study was designed to assess the diversity of *msp-1 *and *msp-2 *among the field isolates from India using allele specific nested PCR assays and sequence analysis. Field isolates were collected from five sites distributed in three states namely, Assam, West Bengal and Orissa.

**Results:**

*P. falciparum *isolates of the study sites are highly diverse in respect of length as well as sequence motifs with prevalence of all the reported allelic families of *msp-1 *and *msp-2*. Prevalence of identical allelic composition as well as high level of sequence identity of alleles suggest a considerable amount of gene flow between the *P. falciparum *populations of different states. A comparatively higher proportion of multiclonal isolates as well as multiplicity of infection (MOI) was observed among isolates of highly malarious districts Karbi Anglong (Assam) and Sundergarh (Orissa). In all the five sites, R033 family of *msp-1 *was observed to be monomorphic with an allele size of 150/160 bp. The observed 80–90% sequence identity of Indian isolates with data of other regions suggests that Indian *P. falciparum *population is a mixture of different strains.

**Conclusion:**

The present study shows that the field isolates of eastern and north-eastern regions of India are highly diverse in respect of *msp-1 *(block 2) and *msp-2 *(central repeat region, block 3). As expected Indian isolates present a picture of diversity closer to southeast Asia, Papua New Guinea and Latin American countries, regions with low to meso-endemicity of malaria in comparison to African regions of hyper- to holo-endemicity.

## Background

Information on the nature and extent of genetic diversity within *Plasmodium falciparum *is essential in understanding the mechanism underlying the pathology of malaria, the acquisition of immunity, the spread of drug resistance and the condition of transmission. Molecular techniques have facilitated the studies on genetic diversity of *Plasmodium *species particularly from field isolates collected directly from patients. Polymerase chain reaction assay has been specially a very useful tool in epidemiological studies. Polymorphic markers could be identified from genomic DNA isolated from small quantities of blood spotted on filter papers. Studies on genetic diversity, the differentiation of different strains within a *Plasmodium *species, presence of multiple parasite strains/types in individual host etc. are reported from different regions of the globe [1-13]. However, limited reports are available on the genetic diversity existing among *P. falciparum *population of India [14-19]. This paper reports polymorphism observed in merozoite surface protein genes (*msp-1 *and *msp-2*) among field isolates of *P. falciparum *collected from north-eastern and eastern regions of India.

Two highly polymorphic and widely used markers are *msp-1 *and *msp-2 *and the large allelic polymorphism has been reported in the block 2 of the *msp-1 *gene and the central repetitive domain (block3) of the *msp-2 *gene. Families differing in nucleotide sequences and in number of repetitive sequences (length variation) were used for genotyping purposes. In *msp-1 *(block 2) three distinct allelic families have been described: K1, MAD20 and RO33 while *msp-2 *has two distinct families, 3D7 (IndoChina) and FC27 [12,20,21].

## Methods

**Figure 1:**
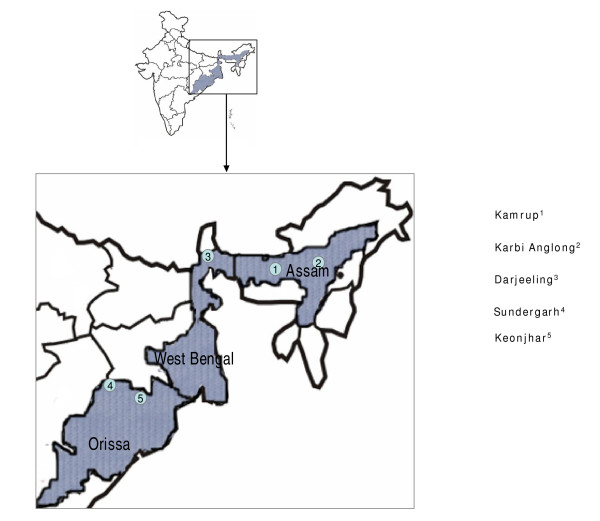
Map of India showing sampling sites.

*P. falciparum *isolates were collected from microscopically diagnosed *P. falciparum *positive subjects in three states with varied malaria epidemiology (Figure 1). Blood was spotted on filter paper strips (Whatman 3 mm) by pricking a finger after obtaining the consent of patient or guardian in case of children. This study has approval of the ethical committee of the National Institute of Malaria Research.

### Study areas

#### Assam

The Assam state contributes 64% of the malaria positive cases and 75% of the *P. falciparum *cases in the north eastern region. The area is highly endemic for malaria and is known for its persistent transmission [22], with the prevalence of multi drug resistant strains. The area is dominated by mongoloid tribes namely Khasis, Bodos and Mikir (Karbi). Samples studied were from two endemic districts Karbi Anglong and Kamrup.

#### West Bengal

An eastern state accounting for about 10% of the total malaria cases in the country and areas along international borders pose a serious problem. The study district Darjeeling, is dominated by Gorkhas (Nepali) population, however, labourers from adjoining state Bihar also frequent the tea gardens. The transmission is seasonal from May to July and then again September to November. *P. falciparum *proportion is in the range of 50–70.

#### Orissa

State located in eastern plateau, contributes 22% of total malaria cases, 43% of falciparum cases and 50% of all reported deaths due to malaria although it constitutes only 4% of the total population of India [23]. *P. falciparum *is the major cause of malaria and accounts for 80–90% of malaria cases. Samples were collected from two malaria endemic districts namely Sundergarh and Keonjhar. Area is dominated by tribals and predominant tribes being Oram, Khadia, Pradhan and Munda. Malaria transmission in District Sundergarh has been reported to be meso to hyper endemic [24].

### Genomic DNA isolation and PCR amplification

Genomic DNA of *P. falciparum *from parasitized blood spots was isolated using QiaAmp DNA minikit as per manufacturer instructions (Qiagen, Hilden, Germany). Primers and PCR protocols were followed as previously described by Snounou *et al *[11] for family specific allele analysis of *msp-1 *(block 2) and *msp-2 *(block 3). PCR amplification was performed on thermal cycler (Perkin Elmer 9700/2400, UK) in a final volume of 20 μl. The PCR products were visualized by UV transillumination at 302 nm on gel documentation system (Syngenta, USA) after electrophoresis on 2% agarose gel (Promega/Boehringer) using 0.5 × TBE buffer at 80–100 volts. Allele sizes were calculated using Genetool programme.

A limited number of isolates representing different families of *msp-1 *and *msp-2 *were sequenced using the Big Dye Terminator cycle-sequencing kit (Applied Biosystems, Foster City, CA) and the ABI Prism 310 automated DNA sequencer (Applied Biosystems). The sequences were then analysed using the DNASTAR software package (DNASTAR, Madison, WI). Sequence data have been submitted to GenBank vide accession numbers DQ485417 to DQ485451.

To understand the identity of Indian isolates with respect to isolates of other regions, sequence data available in public domains were downloaded for allelic families of *msp-1&2 *and details are given below; Thailand(K1-M77730, MAD20-M77722, R033-AAA29684, 3D7-U91676), Vietnam(K1-AF509651, MAD20-AF509653&94, FC27-AF104696, 3D7-AF104693), Tanzania(K1-AF061134, FC27-AY532386), Brazil(K1-AF509714, MAD20-AY714585, FC27-DQ115973, 3D7-AF177389), China(MAD20-AF251345), Sudan(MAD20-AF034635), Iran(MAD20-AY138509, R033-AY138507, FC27-DQ338451), Indonesia(K1-AF191061, R033-AAF18431), Western Africa(R033-PFAMSA1), Kenya(R033-AAM21583), Ghana(FC27-AF329577), PNG(FC27-AF329579), Gambia(FC27-U91668, 3D7-U91665) and Nigeria(3D7-AF148224).

## Results

One hundred and thirty one *P. falciparum *isolates analysed during the study have demonstrated highly diverse nature of field isolates in respect of *msp-1 *(block 2) and *msp-2 *(central repeat region, block3).

All the three reported families of *msp-1*(K1, MAD20 and RO33) and two of *msp-2 *(FC27 and 3D7), were observed among the isolates of all the five study sites (Table 1). Proportion of isolates with K1 family ranged from 33.3% to 72.7% with 6 alleles in the range of 140 to 280 bp. Proportion of isolates with MAD20 family ranged between 13.6% to 72.7% and a total of six alleles were observed within 120 to 240 bp. RO33 proportions ranged from 15.0% to 41.7% and the family was observed to be monomorphic with an allele size of 150/160 bp. Observed proportions, numbers and size range of alleles among the isolates of different study sites are given in Table 2.

**Table 1:** Distribution of size variants of *msp-1 *and *msp-2 *in Indian *P. falciparum *isolates

	**Assam**		**Orissa**		**West Bengal**
	
	**Karbi Anglong**	**Kamrup**	**Keonjhar**	**Sundergarh**	**Darjeeling**
***msp-1***	**n = 18**	**n = 27**	**n = 24**	**n = 40**	**n = 22**
**K1**	1	8	7	17	12
**MAD20**	2	5	7	13	1
**R033**	0	5	8	4	6
**K1+MAD20**	10	3	0	4	2
**K1+R033**	1	3	1	1	1
**MAD20+R033**	0	2	1	1	0
**K1+MAD20+R033**	4	1	0	0	0
**Multiclonal isolates %**	83.3	33.3	8.3	15.0	13.6
***msp-2***	**n = 22**	**n = 28**	**n = 19**	**n = 40**	**n = 22**
**FC27**	4	5	8	9	4
**3D7**	13	21	6	6	18
**FC27+3D7**	5	2	5	25	0
**Multiclonal isolates %**	22.7	7.1	26.3	62.5	0.0

**Table 2:** Observed proportions of various families of *msp-1*&*2*, allele numbers and size range among study isolates.

**Markers**	**Assam**		**Orissa**		**West Bengal**
	
	**Karbi Anglong**	**Kamrup**	**Keonjhar**	**Sundergarh**	**Darjeeling**
***msp-1***					
**K1**					
Obs. Nos. (%)	16 (72.7)	15 (53.6)	8 (33.3)	22 (55.0)	15 (68.2)
Allele					
Obs. Nos.	5	2	3	5	1
Size range (bp)	140–240	170,240	170–240	170–280	140
**MAD20**					
Obs. Nos. (%)	16 (72.7)	11 (39.3)	8 (33.3)	18 (45.0)	3 (13.6)
Allele					
Obs. Nos.	3	3	1	3	1
Size range (bp)	120–220	200–240	220	120–180	120
**RO33**					
Obs. Nos. (%)	5 (22.7)	11 (39.3)	10 (41.7)	6 (15.0)	7 (31.8)
Allele					
Obs. Nos.	1	1	1	1	1
Size range (bp)	150/160	150/160	150/160	150/160	150/160
**MOI**	**2.1**	**1.4**	**1.1**	**1.2**	**1.1**
***msp-2***					
**FC27**					
Obs. Nos. (%)	9 (40.9)	7 (25.0)	13 (68.4)	34 (85.0)	4 (18.2)
Allele					
Obs. Nos.	6	1	1	3	1
Size range (bp)	250–500	300	300	300–400	300
**3D7**					
Obs. Nos. (%)	18 (81.8)	23 (82.1)	11 (57.9)	31 (77.5)	18 (81.2)
Allele					
Obs. Nos.	6	2	3	8	2
Size range (bp)	480–600	450,550	450–500	420–580	450,500
**MOI**	**1.2**	**1.1**	**1.3**	**1.6**	**1.0**

**MOI **– Multiplicity of Infection

In *msp-2*, the reported families FC27 and 3D7 were observed among the isolates of all the 5 study sites (Table 1). Proportion of FC27 family varied from 18.2% to 85.0% and that of 3D7 ranged from 57.9% to 82.1%. Proportional prevalence of FC27 and 3D7 families was significantly different between the study sites (χ^2 ^= 16.5, P = 0.002), particularly between Orissa and Assam (χ^2 ^= 10.20, P = 0.001), Orissa and West Bengal (χ^2 ^= 9.65, P = 0.002). It is seen from Table 2 that in Assam and West Bengal isolates, prevalence of 3D7 family was higher than FC27 family and vice versa in Orissa isolates. Six alleles of FC27 in the range of 250–500 bp and 9 alleles of 3D7 in the range of 420–600 bp were observed (Table 2).

Proportion of multiclonal isolates (multiple infection) in different study sites is given in Table 1 and proportion ranged from 13.6% in Darjeeling, West Bengal to 83.3% in Karbi Anglong, Assam. Among the multiclonal isolates, all possible combinations of *msp-1 *families namely (K1+MAD20, MAD20+R033, K1+R033 and K1+MAD20+R033) were observed and proportion of K1+MAD20 was highest 54.3%(19/35). Complexity of infection (multiplicity of infection, MOI) was highest (2.05) in Karbi Anglong (Assam) and lowest (1.0) in Darjeeling, West Bengal (Table 2). MOI was estimated by dividing the total number of fragments detected in the individual system by the number of samples positive in the particular system (either *msp-1 *or *msp-2*).

**Figure 2:**
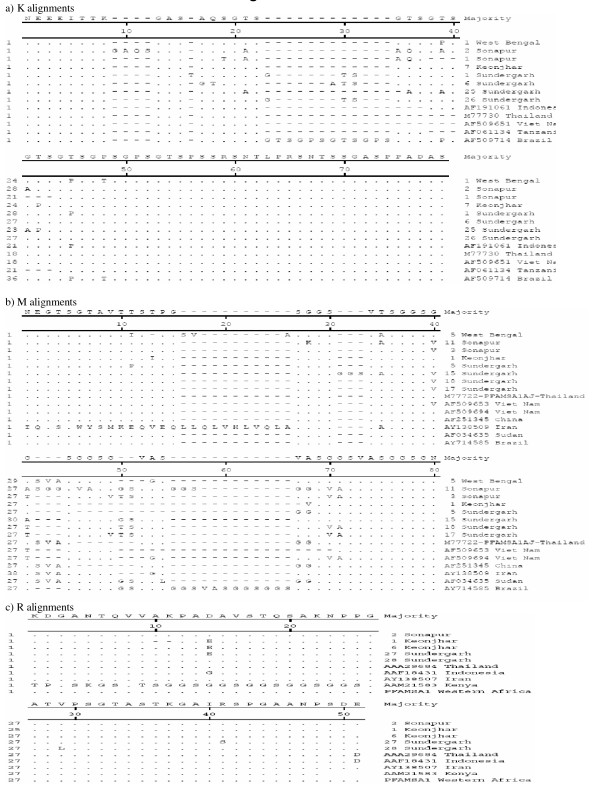
Alignment for the amino acid sequences corresponding to **a) **K1, **b) **MAD20 and **c) **RO33 families of *msp-1*. Sequences shown are either of isolates collected during the study or from the GenBank database. Deletions are represented by minus sign (-) and alphabets represent a change in amino acid at the position.

**Figure 3:**
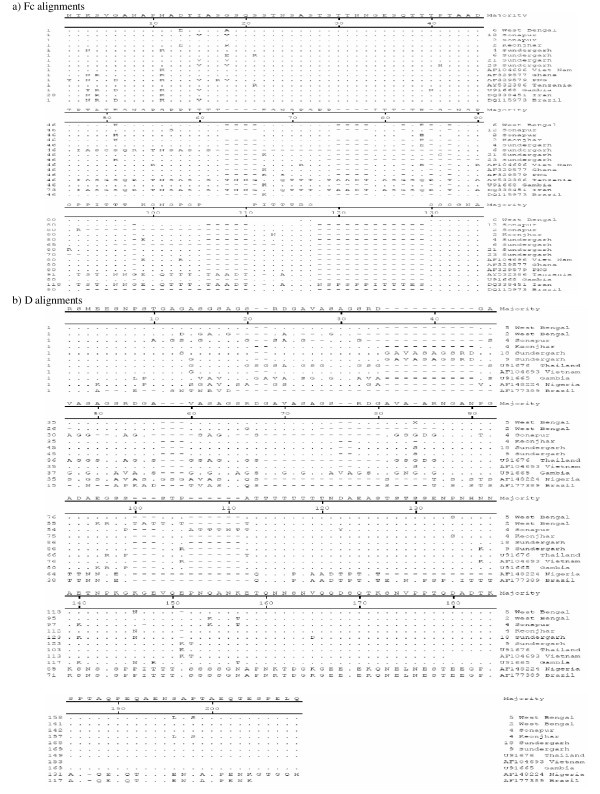
Alignment of the amino acid sequences corresponding to **a) **FC27 and **b) **3D7 families of *msp-2*. Sequences shown are either of isolates collected during the study or from the GenBank database. Deletions are represented by minus sign (-) and alphabets represent a change in amino acid at the position.

Analysis of *msp-1 *and *msp-2 *sequence data revealed above 80% identity of study isolates among themselves in general and above 70% with isolates of other countries with a few exceptions. In K1 family, 88 to 100% similarity was observed with K1 allelic sequences reported for isolates of Indonesia, Thailand, Vietnam, Tanzania and Brazil. Similar identity was observed in MAD20 family with isolates of China, Brazil, Vietnam, Thailand, Sudan and Iran. RO33 allelic sequences of Indian isolates were all very identical and had shown above 94% similarity with sequences reported for isolates of Thailand, Indonesia, Iran and Western Africa except Kenya (Figure 2a–c). Allelic families of *msp-2*, FC27 and 3D7 showed above 70% identity with isolates of Vietnam, Ghana, PNG, Thailand and Gambia. On the other hand, with Iran and Tanzania isolates, identity ranged between 60–70% for Indian FC27 allele and less than 30% with Nigeria and Brazil isolate for 3D7 sequences of the present study (Figures 3a&b).

## Discussion

Observed highly diverse nature of *P. falciparum *isolates of eastern and northeastern regions of India in respect of length as well as sequence motifs with prevalence of all the allelic families of *msp-1 *and *msp-2 *is in agreement with the earlier reports on Indian isolates [16-19,25].

Prevalence of identical allelic composition as well as high level of sequence identity of alleles in five study areas of three states suggest for a considerable amount of gene flow between the *P. falciparum *populations of different states. Labourers from Orissa, Bihar and other parts of West Bengal come to work in tea gardens of Darjeeling (WB) and Assam and their to and fro movement from native place to work place may be facilitating the gene flow. Observed high proportion of multiclonal isolates was in accordance with the reports of other workers [17,18] on Indian isolates. A comparatively higher proportion of multiclonal isolates as well as multiplicity of infection (MOI) was observed among isolates of district Karbi Anglong (Assam) and district Sundergarh (Orissa). Both the districts are highly endemic for malaria with *P. falciparum *proportion being above 80%. Similar observations that the extent of diversity and multiplicity of infection in an area is related to level of malaria endemicity has been made by Ranjit & Sharma [18], Babiker *et al *[26] and Zwetyenga *et al *[27].

Present study for the first time revealed that population structure of *P. falciparum *isolates is identical in two highly malarious regions (Assam and Orissa states) of India as revealed by presence of common allelic composition in both the states as well as high level of identity among allelic sequences of isolates from two areas. However, a higher MOI based on *msp-1 *in Assam and based on *msp-2 *in Orissa, suggests that local factors such as vector population, human host as well as drug susceptibility pattern of the parasites in an area may be playing a role in defining the population structure of the field isolates. A recent study from Iran reports high level of diversity of *msp-1 *and *msp-2 *markers along with high proportion of multiclonal isolates (87%) and MOI (3.06), due to emergence of drug-resistant *P. falciparum *[13].

Distribution of families of *msp-1 *and *msp-2 *and their allelic variations were similar to that reported from other countries with low or meso-endemicity of malaria i.e. Southeast Asia, Latin America and Papua New Guinea [2,4,5,9,24,28,29]. Monomorphic nature of RO33 family of *msp-1 *has also been reported earlier in isolates of other regions including India and observed 150/160 bp was the most commonly reported allele in other continents also [1,7,8,10]. Observed good sequence identity of Indian isolates with data of other regions suggests that Indian *P. falciparum *population is a mixture of different strains. Further studies on the genetic diversity of *P. falciparum *isolates from other regions with varied malaria epidemiology as well as longitudinal studies to understand the clonal fluctuations associated with transmission intensity are important, more so from Indian subcontinent which is a large country with varied malaria paradigm.

## Conclusion

The present study shows that field isolates of eastern and north-eastern regions of India are highly diverse in respect of *msp-1 *(block 2) and *msp-2 *(central repeat region, block 3) with identical population structure and exhibit a level of diversity similar to that in Papua New Guinea, Southeast Asia and South and Central America, regions with low to meso endemicity of malaria.

## Competing interests

The author(s) declare that they have no competing interests.

## Authors' contributions

HJ – Conception & designing of the study, co-ordination of molecular analysis work, analysis & interpretation of data, manuscript preparation. NV – Planning, designing and co-ordination of field studies. AV – Sequencing assays, data analysis & interpretation. AK, PKM, SS, SKP – PCR assays, compilation and analysis of data, drafting of manuscript. SKS, VD, SB, NN, MSM – Epidemiological studies. SKS and APD – Guidance.
